# A systematic review of vaccine-induced thrombotic thrombocytopenia in individuals who received COVID-19 adenoviral-vector-based vaccines

**DOI:** 10.1007/s11239-021-02626-w

**Published:** 2022-02-14

**Authors:** Mostafa H. Elberry, Hussien Ahmed H. Abdelgawad, Aboalmagd Hamdallah, Walid Shaban Abdella, Ahmed Sayed Ahmed, Hazem S. Ghaith, Ahmed Negida

**Affiliations:** 1 Virology and Immunology Unit, Cancer Biology Department, National Cancer Institute, Cairo University, Cairo, Egypt; 2 Clinical Research Management Program, Edson College of Nursing and Health Innovation, Arizona State University, Phoenix, USA; 3 Center for Urban Health Disparities Research and Innovation, Morgan State University, Baltimore, MD USA; 4 Department of Biology, Morgan State University, Baltimore, MD USA; 5 Faculty of Medicine Al-Azhar University, Damietta, Egypt; 6 Faculty of Medicine, Ain Shams University, Cairo, Egypt; 7 Faculty of Medicine Al-Azhar University, Cairo, Egypt; 8 Department of Global Health and Social Medicine, Harvard Medical School, Boston, MA USA; 9 School of Pharmacy and Biomedical Sciences, University of Portsmouth, Portsmouth, UK; 10 Faculty of Medicine, Zagazig University, Sharkia, Egypt

**Keywords:** COVID-19, SARS-CoV-2, Vaccine, AstraZeneca, Johnson and Johnson, ChAdOx1 nCoV-19

## Abstract

Reports of thrombotic response after receiving COVID-19 Adenoviral-Vector Based Vaccines raise concerns about vaccine-induced thrombotic thrombocytopenia (VITT); therefore, we conduct this systematic review to report susceptible demographics outcomes, commonalities, and prognosis of reporting cases. We identified published articles by searching PubMed, SCOPUS, and Web of Science from December 2020 till May 2021, with an updated search in September 2021. All case reports and case series reporting thrombotic response after receiving COVID-19 Adenoviral-Vector Based Vaccines were eligible for including. In addition, two authors independently extracted data and assessed the quality of the included studies. A total of 157 patients with thrombotic events after the ChAdOx1 nCoV-19 vaccine and 16 patients with thrombotic events after Ad26.COV2. S vaccine was included in our study. 72% of the ChAdOx1 nCoV-19 cases were females, while in Ad26.COV2.S subgroup, all reported patients were females. The commonest presentations were deep vein thrombosis 20 (12.7%) and cerebral venous sinus thrombosis 18 (11.5%) in the ChAdOx1 nCoV-19 subgroup while cerebral venous sinus thrombosis 14 (87.5%) and pulmonary embolism 2 (12.5%) in the Ad26.COV2. S subgroup. In this study, we described the certain demographics associated with VITT and the clinical presentations of those cases in the ChAdOx1 nCoV-19 and Ad26.COV2. S vaccines. Young individuals, particularly females, may be more susceptible to VITT, and future studies should seek to confirm this association. In addition, the clinical presentation of VITT commonly includes cerebral thrombi, pulmonary embolism, and deep venous thrombosis, but other presentations are also possible, highlighting the importance of clinical vigilance in recent vaccine recipients.

**Supplementary Information:**

The online version contains supplementary material available at 10.1007/s11239-021-02626-w.

## Highlights

- Thrombotic risk increases in young females after receiving received COVID-19 adenoviral-vector-based vaccines.
- The most common clinical presentations of vaccine-induced thrombotic thrombocytopenia are cerebral thrombi, pulmonary embolism, and deep venous thrombosis.
- Longitudinal head to head studies are needed to confirm the association.

## Introduction

The COVID-19 epidemic as a major public health problem has been associated with increased morbidity and mortality worldwide. The available antiviral drugs and other experimental drugs did not show any significant efficacy against the severe acute respiratory syndrome coronavirus-2 (SARS-CoV-2) [1].

Vaccination against SARS-CoV-2 that minimizes both the rates of infection and serious complications is one of the most effective strategies to prevent and control the current COVID-19 pandemic [2–6]. New vaccines against SARS-CoV-2 have been produced at a rate unprecedented in medical history [7]. In addition, dozens of COVID-19 candidate vaccines have been registered in the clinical trial database (clinicaltrials.gov) [8].

Based on large-scale clinical trial results, the mRNA vaccine (BNT162b2) developed by Pfizer/BioNTech was the first vaccine to be granted FDA approval in December 2020 [4], and other vaccines soon followed in its footsteps following the publication of their trial results. Two vaccines approved by the European Medicines Agency are adenoviral vector-based vaccines (ChAdOx1 nCoV-19, COVID-19 Vaccine AstraZeneca [Vaxzevria] and Ad26.COV2. S, Covid-19 Vaccine Janssen). Although the efficacy and effectiveness of these multiple vaccines have been established, important differences as storage conditions, validity durations, mechanisms of action, number of doses required, and side-effects [9].

Following vaccination with Ad26.COV2. S or ChadOx1 nCoV-19, cases of thrombosis consistent with thrombocytopenia, known as vaccine-induced thrombotic Thrombocytopenia (VITT), have been identified. Many of these cases have been linked to autoantibodies against the platelet factor 4 (PF-4) antigen, which are similar to those observed in patients with autoimmune heparin-induced Thrombocytopenia (HIT) [10–13]. The first of these case reports, to our knowledge, was published on the 8th of April by D’Agostino et al. [14], with a slew of other reports following thereafter. In addition, events have also been reported for Ad26.COV2. S vaccine, with the earliest report being that of See et al. [11].

The importance of these complications is several-fold: first, from a medical perspective, it is important to understand as well as possible a potential complication of a vaccine that is going to be distributed on such a massive scale, both in terms of treatment and for risk–benefit calculations. Second, owing to the potential fatality of some of these complications and the media coverage they’ve received, they may contribute to a significant increase in vaccine hesitancy [15]. This is especially problematic as issues of hesitancy preceded the side-effect reports owing to a general distrust of the pharmaceutical establishment, and such side-effects are only likely to further exacerbate said distrust. Third, because of easier storage conditions, the ChAdOx1 nCoV-19 vaccine is more likely to be consumed in developing countries than alternatives such as mRNA-based vaccines. To that extent, minimizing global disparities due to the COVID pandemic includes the supply of sufficient vaccines to nations in need and the minimization of any adverse effects thereof.

Therefore, this systematic review aims to summarize the reported cases of thrombosis and thrombocytopenia in patients receiving adenoviral vector-based COVID-19 vaccines to identify the susceptible demographics, outcomes, and commonalities in terms of predispositions across the reported cases. Second, reporting on the outcomes of the reported cases.

## Methods

We followed the guidelines of the Preferred Reporting Items for Systematic Reviews and Meta-analysis (PRISMA statement) and the Meta-analysis of Observational Studies in Epidemiology (MOOSE statement) when conducting this systematic review [16].

### Literature search strategy

We searched PubMed, SCOPUS, and Web of Science from December 2020 till May 2021, with an updated search on September 2021, for relevant articles reporting the thromboembolic events after Adenovirus vector-based vaccines using the following Keywords: “ChAdOx1 nCoV-19 vaccine”, “AstraZeneca Vaccine” “Ad26.COV2. S”, “Johnson & Johnson Vaccine”, “COVID-19”, “SARS-CoV-2”, “Thrombosis”, and “Thrombocytopenia” as seen in Supplementary File 1.

### Eligibility criteria and Study selection

Studies achieving the following PIOS criteria were included:

- Population: Reports of individuals who developed thrombosis and thrombocytopenia associated with ChAdOx1 nCoV-19 or Ad26.COV2. S vaccines administration
- Indicator (or risk factor): ChAdOx1 nCoV-19 vaccine or Ad26.COV2. S administration
- Outcome: Reports of individuals who developed post-vaccination thrombosis and thrombocytopenia.
- Study design: Articles that were described as case reports and case series

We excluded animal, laboratory, in vitro studies, conferences, reviews, and book chapters. Two authors independently screened the retrieved citations, and this was performed in two steps: title and abstract screening, followed by full-text screening. Finally, we examined the reference lists of included articles to identify additional studies. Any disagreements were resolved by discussion.

### Assessing the risk of bias

Two reviewers independently assessed the quality of the eligible articles using the appropriate quality assessment tools, which depend on the study design of each of the included articles. For the case series, we used the NIH Quality Assessment Tool for case series [17], and the Joanna Briggs Institute (JBI) checklist to appraise the case reports [18]. The NIH criteria assess studies based on the following domains: clarity of objectives, clarity of study population and case definitions, whether the cases were consecutive and comparable, whether interventions were described clearly, the reliability and validity of outcome measurement, adequacy of follow-up length, statistical methodology, and whether results were well-described. The JBI checklist assesses the following: clarity of patient demographics, clinical history and presentation, diagnostic assessments, treatments, condition post-treatment, adverse events, and takeaway points.

### Data extraction

We extracted the data from each included study using a pre-specified uniform data extraction sheet. The extracted data included the following domains: the number of patients included, demographic data on the country, age and sex, the type of vaccine received, clinical features including comorbidities, time to admission, and symptoms on admission, laboratory findings including coagulation parameters (PT/aPTT), platelet count, hemoglobin, D-dimers, fibrinogen levels, antiplatelet factor 4 assays, the location of thrombotic events, management including the choice of medications (steroids, IVIG, heparin coagulation, non-heparin anticoagulants), and outcomes on recovery or death.

## Results

### Literature Search

**Fig. 1:**
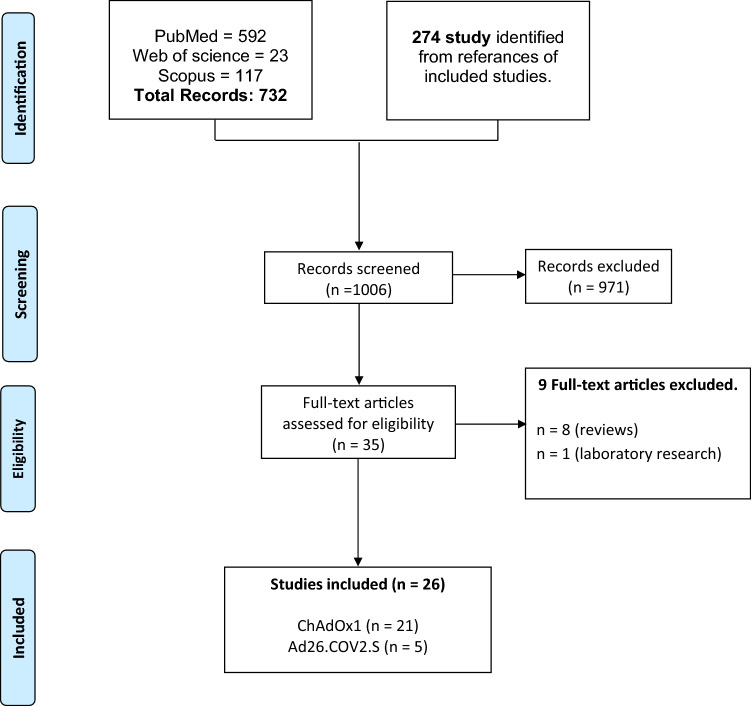
Shows the PRISMA flow chart which summarizes the literature search, and included studies

A total of 1006 records were identified in the initial literature search and reference lists of included articles. Records were screened by titles and abstracts, and 971 articles were excluded. Thirty-five articles were retrieved for full-text evaluation. Twenty-six articles that met our criteria were included in this systematic [10–13, 19–40] (see PRISMA flow diagram, Fig. 1).

### Characteristics of cases among the included studies

This systematic review included case report (n = 16), and case series (n = 10) described cases developing events of thrombosis and thrombocytopenia after administration of ChAdOx1 nCoV-19 vaccine (n = 157), or Ad26.COV2. S vaccine (n = 16). Most cases (72% and 100% in the ChAdOx1 and Ad26.COV2. S cohorts, respectively) were female. The overall age was relatively low (mean of 43.2 years in the ChAdOx1 cohort, 8 (50%) patients were below 40 in the Ad26.COV2. S cohort). The average time from vaccination to admission was 10.5 and 15.9 days in the ChAdOx1 and Ad26.COV2. S cohorts, respectively. The summary of included studies is presented in Table 1, and a descriptive summary of included cases is presented in Table 2.

**Table 1:** Shows a description of each patient in the included case series and case reports

Study ID	Design	No. of patient	Country	Patient	Age, year	Gender	Comorbidities/Pre-existing conditions	Vaccine	Time from vaccination to admission	Symptoms on Admission	Thromboembolic events (Site of thrombosis)
Agostino 2021	Case report	1	Italy	#1	54	Female	Meniere’s disease	Chadox1 ncov-19	12	Acute cerebrovascular accident	Brain: right frontal, the temporal lobes and superior sagittal sinus Thorax: left upper lobe segmental branches, of left interlobar artery, of the right middle lobe segmental branches and of the right interlobar artery Abdomen: left portal branch and at the level of right supra hepatic vein
Bayas 2021	Case report	1	Germany	#1	55	Female	–	Chadox1 ncov-19	10	Conjunctival congestion Retro-orbital pain Diplopia	Superior ophthalmic vein
Blauenfeldt 2021	Case report	1	Denmark	Day 1	60	Female	Hashimoto’s thyroiditis Hypertension	Chadox1 ncov-19	7	Persistent abdominal pain Left-sided weakness Eye deviation to the right	Right internal carotid artery
				Day 2							
				Day 3							
				Day 6							
Greinacher 2021	Case series	11	Germany and Austria	#1	Mean (SD) = 35.7 (7.8)	Females (n = 9) Males(n = 2)	–	Chadox1 ncov-19	10	Chills Fever Nausea Epigastric Discomfort	CVST Splanchnic-vein thrombosis Pulmonary embolism Aortoiliac
				#2			–	Chadox1 ncov-19		–	Pulmonary embolism
				#3			–	Chadox1 ncov-19		–	CVST
				#4			Chronic neurologic disorder	Chadox1 ncov-19		–	CVST
				#5			Von Willebrand disease	Chadox1 ncov-19		–	CVST Splanchnic-vein thrombosis Pulmonary embolism Right intraventricular Iliofemoral vein Inferior Vena Cava thrombosis
				#6			–	Chadox1 ncov-19		–	CVST
				#7			–	Chadox1 ncov-19		–	CVST
				#8			–	Chadox1 ncov-19		–	CVST Widespread microvascular (brain, lungs, kidneys)
				#9			–	Chadox1 ncov-19		–	CVST Multiple organ thrombi
				#10			–	Chadox1 ncov-19		–	CVST Splanchnic-vein thrombosis
				#11			–	Chadox1 ncov-19		–	Cerebral hemorrhage
Kasuistik 2021	Case series	2	Norway	#1	30th	Male	–	Chadox1 ncov-19	14	Back pain Fever Frostbite	DVT
				#2	40th	Male	–	Chadox1 ncov-19	14	Joint pain Felt exhausted Dyspnea with light exertion	DVT
Muster 2021	Case report	1	Austria	#1	51	Female	–	Chadox1 ncov-19	11	Dyspnea Fatigue Cough	Central pulmonary embolism Left internal iliac vein Common iliac vein Inferior vena cava
Schultz 2021	Case series	5	Norway	#1	37	Female	Pollen allergy	Chadox1 ncov-19	8	Fever Headache Visual disturbances	Cortical veins Left transverse sinus Sigmoid left sinus
				#2	42	Female	Pollen allergy	Chadox1 ncov-19	10	Headaches Drowsiness	Cortical veins Left transverse sinus Left sigmoid sinus
				#3	32	Male	Asthma	Chadox1 ncov-19	7	Back pain	Portal vein, left hepatic vein, splenic vein, azygos, vein, hemiazygos vein, and several basivertebral veins
				#4	39	Female	–	Chadox1 ncov-19	10	Headaches Abdominal pain	Inferior sagittal sinus, vein of Galen, straight sinus, right transverse sinus, and right sigmoid sinus
				#5	54	Female	Hypertension	Chadox1 ncov-19	7	Headaches Hemiparesis	Cortical veins, superior sagittal sinus, both transverse sinuses, and left sigmoid sinus
Scully 2021	Case series	23	United Kingdom	#1	30	Female	–	Chadox1 ncov-19	13	–	CVT Portal Vein thrombosis Pulmonary embolism Ischemic bowel with infarction
				#2	55	Female	–	Chadox1 ncov-19	6	–	Portal Vein thrombosis Acute atherosclerotic thrombosis Intracerebral hemorrhage
				#3	26	Female	–	Chadox1 ncov-19	12	–	CVT
				#4	52	Female	–	Chadox1 ncov-19	10	–	Post mortem: thrombosis in the lungs, intestine, CVT, and Intracerebral hemorrhage
				#5	38	Male	–	Chadox1 ncov-19	14	–	Extensive bilateral pulmonary embolism with heart strain
				#6	49	Female	–	Chadox1 ncov-19	15	–	CVT Internal Jugular vein thrombosis Subarachnoid hemorrhage
				#7	25	Male	–	Chadox1 ncov-19	9	–	CVT
				#8	32	Male	–	Chadox1 ncov-19	19	–	CVT
				#9	35	Female	–	Chadox1 ncov-19	9	–	CVT
				#10	77	Male	–	Chadox1 ncov-19	8	–	Pulmonary embolism
				#11	66	Male	–	Chadox1 ncov-19	12	–	DVT Adrenal hemorrhage
				#12	34	Male	–	Chadox1 ncov-19	14	–	CVT
				#13	54	Male	–	Chadox1 ncov-19	10	–	Portal Vein Thrombosis Myocardial infarction
				#14	71	Female	–	Chadox1 ncov-19	14	–	Hemorrhagic symptoms only
				#15	22	Female	–	Chadox1 ncov-19	10	–	CVT Intracerebral hemorrhage
				#16	39	Female	–	Chadox1 ncov-19	10	–	MCA infarction
				#17	17	Female	–	Chadox1 ncov-19	17	–	Pulmonary embolism (saddle embolism) with cardiac arrest DVT in the leg
				#18	10	Male	–	Chadox1 ncov-19	10	–	MCA infarction
				#19	14	Female	–	Chadox1 ncov-19	14	–	CVT
				#20	12	Female	–	Chadox1 ncov-19	12	–	CVT
				#21	14	Male	–	Chadox1 ncov-19	14	–	CVT
				#22	24	Female	–	Chadox1 ncov-19	24	–	Pulmonary embolism
				#23	10	Female	–	Chadox1 ncov-19	10	–	CVT
Thaler 2021	Case report	1	Austria	#1	62	Female	Hypothyroidism	Chadox1 ncov-19	8	Flulike symptoms including aching joints, moderate headache, and moderate dizziness	CVT Splanchnic vein thrombosis
Tiede 2021	Case series	5	Germany	#1	63	Female	–	Chadox1 ncov-19	11	Headache Somnolence Dysphasia Right sided hemiparesis Arterial hypertension	CVST Thrombotic microangiopathy
				#2	67	Female	–	Chadox1 ncov-19	8	Headache	Arterial cerebral embolism
				#3	41	Female	–	Chadox1 ncov-19	5	Headache Diplopia	Transient ischemic attack
				#4	61	Female	–	Chadox1 ncov-19	9	Fatigue	Superficial vein thrombosis
				#5	61	Female	–	Chadox1 ncov-19	9	Headache Dysarthria Left sided hemiplegia Conjugated gaze palsy	Arterial cerebral thrombosis Popliteal artery thrombosis
Tobaiqy 2021	Case series	28	Several European countries	28	18–64 years = 3 (10.7%) 65–85 years = 9 (32.1%) > 85 years 16 (57.1%)	9 males 19 females	–	Chadox1 ncov-19	–	–	CVST (n = 1) pulmonary embolism (n = 6) Carotid artery thrombosis (n = 1) Peripheral artery thrombosis (n = 1) Pelvic vein thrombosis (n = 2) DVT (n = 16) Thrombophlebitis (n = 2) Thrombosis (n = 5)
Wolf 2021	Case series	3	Germany	#1	22	Female	–	Chadox1 ncov-19	–	Generalized epileptic seizures	CVST
				#2	46	Female	–	Chadox1 ncov-19	–	Mild aphasia Homonymous hemianopia to the right	CVST
				#3	36	Female	–	Chadox1 ncov-19	–	Aphasia Reduced consciousness	CVST
Schulz 2021	Case series	62	Germany	Chadox1 ncov-19 53 (85.5%)	Mean (SD) = 46.6 (17.1)	15 males 47 females	Coronary heart disease 2 (3.4%)	Chadox1 ncov-19	Mean (SD) = 10.75 (7.28)	–	CVT Ischemic stroke Intracerebral hemorrhage
Hocking 2021	Case report	1	Australia	#1	44	Male	Previous thrombosis Depression	Chadox1 ncov-19	8	Fevers Fatigue Abdominal discomfort Increased bowel frequency Vague abdominal pains	Portal, splenic, and superior mesenteric vein thrombosis
Umbrello 2021	Case report	1	Italy	#1	36	Female	Upper abdominal pain	Chadox1 ncov-19	–	Fever Asthenia Diffuse osteoarticular pain	Portal, splenic, and mesenteric vein thrombosis
Walter 2021	Case report	1	Germany	#1	31	Male	–	Chadox1 ncov-19	8	Headache Aphasia Hemiparesis	Carotid artery thrombosis
Wiedmann 2021	Case series	6	Norway	#1	34	Female	Pollen allergy	Chadox1 ncov-19	10	Headache Left-sided limb weakness Dysarthria	Right-sided parenchymal and subarachnoid hemorrhage
				#2	42	Female	Pollen allergy	Chadox1 ncov-19	10	Headache Nausea Vomiting Right sided hemiparesis	CVST Cortical vein thrombosis
				#3	37	Female	Pollen allergy	Chadox1 ncov-19	8	Headache Fever Transient numbness in the right foot Right sided visual disturbance	CVST Cortical vein thrombosis
				#4	39	Female	–	Chadox1 ncov-19	7	Headache Abdominal pain	Pulmonary emboli Thrombosis in uterine veins
				#5	54	Female	Hypertension	Chadox1 ncov-19	7	Numbness of her left-sided limbs Headaches Nausea Left sided weakness	Venous infarction Parenchymal hemorrhage Subarachnoid hemorrhage
				#6	–	Male	–	Chadox1 ncov-19	–	–	–
Suresh 2021	Case report	1	UK	1	27	Male	–	ChAdOx1 nCOV-19	2	Intermittent headaches associated with eye floaters Vomiting	CVST
Guan 2021	Case report	1	Taiwan	1	52	Male	–	ChAdOx1 nCov-19	5 days	Nausea Thunderclap headache Pain on the left side of the neck	CVST Left transverse sinus thrombosis sigmoid sinuses thrombosis Left internal jugular vein thrombosis
Costentin 2021	case report	1	France;	1	26	Female	–	ChAdOx1 nCov-19	3	Nausea, Muscle and body aches Fatigue Bilateral progressive headache	left middle cerebral artery thrombosis Pulmonary embolism Portal vein thrombosis
FANNI 2021	case report	1		1	58	Male	–	ChAdOx1 nCov-19	13	Abdominal pain Diarrhea and Vomiting	Portal vein thrombosis Splenic vein Portal vein thrombosis Several branches of the superior mesenteric vein Portal vein thrombosis
Muir 2021	Case report	1	Nebraska	#1	48	Female	–	Ad26.COV2. S	14	Malaise Abdominal pain	CVST (involving the right transverse and straight sinuses)
See 2021	Case series	12	United states	#1	≥ 40	Female	–	Ad26.COV2. S	11	Headache Lethargy	CVST (Right transverse sinus and right sigmoid sinus)
				#2	18–39	Female	–	Ad26.COV2. S	16	Headache	CVST (Left transverse sinus, left sigmoid sinus, confluence of sinuses, and straight sinus)
				#3	18–39	Female	–	Ad26.COV2. S	17	Headache Fever Vomiting	CVST (Superior sagittal sinus, inferior sagittal sinus, straight sinus, cortical veins)
				#4	18–39	Female	–	Ad26.COV2. S	16	Headache Nausea Myalgia Chills Fever	CVST (Right transverse sinus and right sigmoid sinus)
				#5	18–39	Female	–	Ad26.COV2. S	18	Chills Dyspnea Fever Headache	CVST (Right transverse sinus and right sigmoid sinus)
				#6	≥ 40	Female	–	Ad26.COV2. S	15	Back pain Bruising Abdominal pain	CVST (Right transverse sinus and straight sinus)
				#7	18–39	Female	–	Ad26.COV2. S	18	Headache Neck pain Nausea Vomiting Photophobia	CVST (Superior sagittal sinus, transverse sinuses, straight sinus, possible sigmoid)
				#8	18–39	Female	–	Ad26.COV2. S	23	Headache	CVST (Right transverse sinus, right sigmoid sinus)
				#9	≥ 40	Female	–	Ad26.COV2. S	11	Headache Cognitive fogginess Right arm weakness	CVST (Superior sagittal sinus, bilateral cortical veins)
				#10	18–39	Female	–	Ad26.COV2. S	10	Headache Nausea Vomiting Photophobia	CVST (Superior sagittal, right transverse and sigmoid sinus)
				#11	18–39	Female	–	Ad26.COV2. S	25	Headache Blurry vision	CVST (Torcula, bilateral transverse sinus, right sigmoid sinus)
				#12	≥ 40	Female	–	Ad26.COV2. S	13	Headache Petechial rash Neck pain Photophobia Body aches	CVST (Left transverse and sigmoid)
Costello 2021	Case report	1	Colorado	#1	40	Female	Migraines Obesity	Ad26.COV2. S	6	Sudden Headache Body aches fever Chills	CVST Pulmonary embolism
Clark 2021	Case report	1	United states	#1	40	Female	–	Ad26.COV2. S	5	Headache Sinus pressure Myalgias Sore throat Tonsillar exudate	CVST Left internal jugular vein thrombosis Pulmonary emboli
Yocum 2021	Case report	1	United states	#1	62	Female	–	Ad26.COV2. S	37	Altered mental status Spontaneously moving all extremities	Thrombotic thrombocytopenic purpura

Study ID	Lab investigations							Anticoagulation treatment	Other treatment	End outcome
	Hemoglobin g/dl	Platelet count (per mm3)	D-dimer (mg/liter)	Fibrinogen (g/l)	Anti-platelet factor 4 antibodies	PT	aPTT			
Agostino 2021	8.7	–	Elevated	Normal	–	–	41	–	–	Fatal
Bayas 2021	–	30,000	–	–	Negative	–	–	Heparin then switched to phenprocoumon	Dexamethasone 40 mg daily was given for 4 days levetiracetam and lacosamide was given to control the seizure	Recovered
Blauenfeldt 2021	14	118,000	–		Positive	–	–	Post-operative dalteparin 5000 IU	Hydrocortisone 100 mg three times daily Cefuroxime was initiated Platelet inhibitor treatment was deferred due to the possibility of malignant media infarction with subsequent surgery	Fatal
	13.7	50,000	41.8	3.7	Positive	–	28			
	13.5	24,000	97.8	2.7	Positive	–	32			
	8.4	50,000	106.2	2.3	Positive	–	27			
Greinacher 2021	–	13,000	142	0.8	–	–	41.6	Heparin	–	Fatal
	–	107,000	1.8	5.7	–	–	29	LMWH	–	Recovering
	–	60,000	13	–	–	–	–	–	–	Unknown
	–	9000	–	–	–	–	46.6	Heparin	–	Fatal
	–	23,000	–	1.7	–	–	64.8	Heparin	–	Recovering
	–	75,000	2.6	–	–	–	23	–	–	Recovering
	–	29,000	> 33	2.1	–	–	45	Heparin	–	Recovering
	–	16,000	–	–	–	–	–	–	–	Fatal
	–	13,000	21	0.4	–	–	46.1	–	–	Fatal
	–	8000	> 35	0.8	–	–	–	–	–	Fatal
	–	–	–	–	–	–	–	–	–	Fatal
Kasuistik 2021	12.7	334,000	< 0.4	–	–	–	–	Rivaroxaban	–	Recovered
	15.9	303,000	< 0.4	–	–	–	–	Apiksaban 10 mg × 2 for the first 7 days, then 5 mg × 2 for 3 months	–	Recovered
Muster 2021	–	37,000	> 34	Normal	–	Normal	Normal	LMWH	Dexamethasone 40 mg orally	Recovered
Schultz 2021	–	22,000	> 35	2.1	3.66	–	25	Initial low dose of LMWH	–	Fatal
	–	14,000	> 35	0.8	3.44	–	31	Reduced dose of LMWH	Methylprednisolone (1 mg/kg) IVIG (1 g/kg)	Fatal
	–	10,000	> 35	2.3	3.63	–	25	Reduced dose of LMWH	Prednisolone (1 mg/kg) IVIG (1 g/kg)	Recovered
	–	70,000	13	1.2	3.83	–	25	Reduced dose of LMWH	Prednisolone (1 mg/kg) IVIG (1 g/kg)	Recovered
	–	19,000	> 35	1.2	2.93	–	29	Heparin (5000 IU)	Methylprednisolone (1 mg/kg) IVIG (1 g/kg)	Fatal
Scully 2021	–	27,000	16,280	2.5	–	12.1	35	–	–	Recovered
	–	11,000	26,689	1.1	–	13.1	31	–	–	Fatal
	–	64,000	> 5000	3.2	2.45	12.1	34.1	–	–	Recovered
	–	31,000	37,250	1.2	2.26	15	35	–	–	Fatal
	–	16,000	45,229	1.2	2.84	12.8	30.8	–	–	Fatal
	–	14,000	39,049	1.3	–	15.4	36	–	–	Recovered
	–	19,000	–	1.3	–	13.2	34.1	–	–	Fatal
	–	87,000	–	1.7	–	14.1	26.7	–	–	Recovered
	–	65,000	10,316	2.2	–	13.2	28.7	–	–	Recovered
	–	–	6018	2.6	–	13.1	23	–	–	Recovered
	–	34,000	10,388	2.1	–	–	–	–	–	Recovered
	–	23,000	37,000	0.7	Positive	14.8	22	–	–	Recovered
	–	71,000	80,000	1.2	0.76	13.5	32.7	–	–	Fatal
	–	17,000	> 20,000	0.8	Positive	15.4	40.3	–	–	Recovered
	–	100,000	> 10,000	3	1.4	11.1	23.6	–	–	Fatal
	–	57,000	> 5000	4.4	1.4	13.2	27.9	–	–	Recovered
	–	28,000	> 5000	3.8	Positive	12.1	43.4	–	–	Recovered
	–	113,000	22,903	1	2.8	14.3	24.8	–	–	Recovered
	–	7000	31,301	1.1	> 3.00	12.1	34.1	–	–	Recovered
	–	98,000	6574	< 0.4	2.17	16.5	52.7	–	–	Recovered
	–	16,000	62,342	1.2	2.45	13.2	31	–	–	Recovered
	–	61,000	71,859	4.5	> 3.00	14.3	31	–	–	Recovered
	–	36,000	> 20,000	0.7	Negative	15.4	43.4	–	–	Recovered
Thaler 2021	14.7	26,000	52.66	0.84	Strongly positive	26	38.7	Non-heparin anticoagulation	Prednisolone (0.75 mg/kg) High dose IVIG	Recovered
Tiede 2021	–	27,000	> 35.2	–	Strongly positive	–	–	Full-dose unfractionated heparin	Dexamethasone pulse for 4 days Eculizumab (900 mg weekly)	Recovering
	–	40,000	> 35.2	–	Strongly positive	–	–	Argatroban	Dexamethasone pulse for 4 days IVIG, 1 g/kg on 2 consecutive days	Recovered
	–	105,000	22.4	–	Strongly positive	–	–	–		Recovered
	–	12,000	> 35.2	–	Strongly positive	–	–	–	IVIG, 1 g/kg on 2 consecutive days	Recovering
	–	62,000	> 35.2	–	Strongly positive	–	–	Argatroban	Dexamethasone pulse for 4 days IVIG, 1 g/kg on 2 consecutive days	Recovering
Tobaiqy 2021	–	–	–	–	–	–	–	–	–	Not recovered (n = 6) Recovered (n = 3) Recovering (n = 11) Fatal (n = 3) Unknown (n = 5)
Wolf 2021	–	75,000	2.9	–	Positive	–	–	LMWH	–	Recovered
	–	60,000	22.8	–	Positive	–	–	LMWH	–	Recovered
	–	92,000	2.12	–	Positive		–	LMWH	–	Recovered
Schulz 2021	–	–	–	–	–	–	–	Plasmapheresis 2 (3.3%)	IVIG 20 (32.8%) Corticosteroids 4 (6.6%)	–
Hocking 2021	–	70,000	–	–	Strongly positive	–	–	Fondaparinux 10 mg subcutaneous	IVIG at 1 g per kg per day	–
Umbrello 2021	–	–	–	0.5	Positive	13	–	Unfractioned heparin	IVIG at 0.4 g/kg	Recovering
Walter 2021	–	217,000	1.1	2.7	Positive	–	27.5	LMWH Aspirin	–	Recovered
Wiedmann 2021	13.6	33,000	16.2	1.9	2.8	–	28	–	–	Fatal
	12.1	14,000	> 35	0.7	3.5	–	31	–	–	Fatal
	11.4	22,000	> 35	1.8	3.7	–	28	–	–	Fatal
	12.6	70,000	13	1.2	3.8	–	25	–	–	Recovering
	9.6	19,000	> 35	1.1	2.9	–	25	–	–	Fatal
	–	–	–	–	–	–	–	–	–	–
Suresh 2021	15	90,000	34	1.9	Positive	–	27.5	Idarucizumab	High-dose steroids Proton pump inhibitors, IVIG Decompressive craniotomy	Recovered
Guan 2021	–	99,000	> 20	–	Positive	–	–	–	–	Recovering
Costentin 2021	–	57,000	–	0.9	Positive	–	–	–	–	
FANNI 2021	6.8	28,000	39	1	–	–	–	–	–	Fetal
Muir 2021	–	13,000	117.5	0.9	Strongly positive	–	–	Unfractionated heparin	–	–
See 2021	–	12,000	> 20.0	0.9	Positive (n = 11) Negative (n = 1)		31	Heparin treatment (n = 6) did not receive anticoagulation (n = 2)	IVIG (n = 7) Systemic corticosteroids (n = 3)	Fatal (n = 3) Continued ICU care (n = 3) Continued non-ICU hospitalization (n = 2) Discharged home (n = 4)
	–	69,000	1.1	1.6			22.3			
	–	18,000	8.46	0.8			31.1			
	–	127,000	5.45	2.4			31.2			
	–	10,000	7.05	1.4			18.1			
	–	13,000	112.07	0.6			34.5			
	–	64,000	7.84	0.8			Negative			
	–	90,000	6.7	2.4			28			
	–	15,000	> 4	3.3			26.9			
	–	9000	13.47	1.3			24.1			
	–	102,000	41.71	2.1			30.2			
	–	20,000	45.57	1.5			26.4			
Costello 2021	–	20,000	45.5	-	Positive	–	–	A non-heparin anticoagulant, bivalirudin (Angiomax)	Prednisone, 1 mg per kg per day IVIG at 1 g per kg per day	
Clark 2021	15.1	20,000	13.5	1.5	Negative	16		A non-heparin anticoagulant, bivalirudin (Angiomax)	–	Recovering
Yocum 2021	14	29,000	–	1.2	Negative	Normal	26.4	–	High dose steroids	Recovering

*aPTT* activated partial thromboplastin time, *CVST* Cerebral venous sinus thrombosis, *CVT* Cerebral venous thrombosis, *DVT* Deep vein thrombosis, *IVIG* Intravenous immunoglobulin, *LMWH* Low-molecular-weight heparin, *MCA* Malignant middle cerebral artery, *PT* prothrombin time, *SD* Standard deviation. Recovered: means that the patient fully recovered and stop treatment, while recovering means that the patients in a state of improvement, but he is still receiving treatment, whether at home or in the hospital

**Table 2:** Shows the overall descriptive summary of included cases

	ChAdOx1	Ad26.COV2. S
Patient No.	157	13
Gender		
Male n (%)	44 (28%)	0
Female n (%)	113 (72%)	16 (100%)
Age years, mean (SD)	mean (SD) = 43.2 (16.7)) [n = 129] 18–39 (41 patient) 40–60 (81 patient) ≥ 60 (35 patient)	18–39 (8 patients) ≥ 40 (8patients)
Comorbidities/preexisting conditions		
Reported Comorbidities n (%)*	21 (13.4%)	2 (12.5%)
Hypertension	3 (61.9%)	0
Chronic neurologic disorder	1 (4.8%)	0
Asthma	1 (4.8%)	0
Pollen allergy	5 (23.8%)	0
Meniere’s disease	1 (4.8%)	0
Hypothyroidism	1 (4.8%)	0
Hashimoto’s thyroiditis	1 (4.8%)	0
von Willebrand disease	1 (4.8%)	0
Coronary heart disease	2 (9.5%)	
Migraines	0	1 (50%)
Obesity	0	1 (50%)
Other	5 (23.8%)	
Not reported Comorbidities n (%)	136 (86.6%)	14 (87.5%)
Time from vaccination to admission days, mean (SD)	10.5 (5.8) [n = 126]	15.9 (7.8)
Symptoms on Admission n (%)		
Reported symptom n (%)**	33 (21%)	16 (100%)
Headache	18 (54.5%)	13 (81.3%)
Eye symptoms	9 (27.3%)	5 (31.3%)
Fever and Chills	7 (21.2%)	7 (43.8%)
Back pain	2 (6%)	1 (6.3%)
Fatigue	4 (12.1%)	0
Joint pain	3 (9%)	0
Cough	1 (3%)	0
Frostbite	1 (3%)	0
Nausea/Vomiting	8 (24.2%)	6 (37.5%)
Hemiparesis	4 (12.1%)	0
Dyspnea	2 (6%)	0
Abdominal pain	7 (21.2%)	2 (12.5%)
Aphasia, dysphasia, or dysarthria	6 (18.1%)	0
Reduced consciousness	2 (6%)	1 (6.3%)
Dizziness	3 (9%)	0
Left sided hemiplegia	1 (3%)	0
Arterial hypertension	1 (3%)	0
Generalized epileptic seizures	1 (3%)	0
Acute cerebrovascular accident	1 (3%)	0
Lethargy/weakness/body aches	7 (21.2%)	8 (50%)
Not reported symptoms	124 (79%)	0
Thromboembolic events n (%)***		
Cerebral venous sinus thrombosis	18 (11.5%)	14 (87.5%)
Cerebral venous thrombosis	15 (9.6%)	1 (6.3%)
Deep vein thrombosis	20 (12.7%)	–
Pulmonary embolism	16 (10.1%)	2 (12.5%)
Cortical veins	3 (1.9%)	–
Portal Vein thrombosis	6 (3.8%)	–
Carotid artery thrombosis	3 (1.9%)	–
Splanchnic-vein thrombosis	4 (2.5%)	–
Cerebral hemorrhage	9 (5.7%)	–
Malignant middle cerebral artery (MCA) infarction	2 (1.3%)	–
Inferior vena cava	2 (1.3%)	–
Other	70 (44.6%)	1 (6.3%)
Laboratory finding		
Hemoglobin g/dl mean (SD)	12.1 (2.7) [n = 12]	14.5 (0.8) [n = 2]
Platelet count cell per mm3 median (Range)	33,500 (7000–334,000) [n = 62]	20,000 (9000–127,000) [n = 16]
D-dimer mg/l n (%)	Elevated 52 (33.1%) Not reported 103 (65.6%) Normal 2 (1.3%)	Elevated 15 (93.7%) Not reported 1 (6.3%)
D-dimer mg/l median (Range)	26 (0.4–142) [n = 53]	13.5 (1.1–117.5) [n = 15]
Fibrinogen (g/liter) median (Range)	1.2 (0.4–5.7) [n = 46]	141 (59–332) [n = 15]
Antibodies to Platelet Factor 4 n (%)	Positive 39 (24.8%) Negative 2 (1.3%) Not reported 116 (73.9%)	Positive 13 (81.2%) Negative 3 (18.8%)
Pt mean (SD)	14.1 (2.9) [n = 24]	-
APTT mean (SD)	33 (9) [n = 43]	27.5 (18.1–34.5) [n = 12]
Treatment n (%)****		
Reported treatments	48 (30.6%)	16 (100%)
Heparin therapy	20 (41.6%)	7 (43.8%)
Non-heparin anticoagulation	8 (16.7%)	2 (12.5%)
Corticosteroids	18 (37.5%)	5 (31.3%)
Intravenous immunoglobulin	29 (60.4%)	8 (50%)
Not reported any treatment	109 (69.4%)	0
End outcome n (%)		
Recovered	34 (21.7%)	4 (25%)
Recovering	21 (13.4%)	7 (43.8%)
Not recovered	6 (3.8%)	–
Fatal	25 (15.9%)	3 (18.8%)
Unknown	71 (45.2%)	2 (12.5%)
Heparin therapy		
Fatal	6/20 (30%)	–
Recovered	8/20 (40%)	–
Recovering	5/20 (25%)	–
Non-heparin anticoagulation		
Fatal	0/8	–
Recovered	5/8 (62.5%)	–
Recovering	1/8 (12.5%)	–
Corticosteroids		
Fatal	3/18 (16.7%)	–
Recovered	8/18 (44.4%)	–
Recovering	3/18 (16.7%)	–
IVIG		
Fatal	2/29 (6.9%)	–
Recovered	5/29 (17.2%)	–
Recovering	1/29 (3.4%)	–

*aPTT* activated partial thromboplastin time, *PT* prothrombin time, *SD* Standard deviation

(*): any patient might have one or more comorbidities, (**): any patient might have one or more symptoms, (***): any patient might have one or more Thromboembolic events, (****): any patient might receive one or more treatments

### Symptoms at Admission and Pre-existing conditions

On admission, various symptoms were present in 49 patients (33 patients in ChAdOx1 nCoV-19 and 16 patients in Ad26.COV2. S), including headache, eye symptoms, fever, back pain, epigastric pain, and nausea/vomiting. In addition, our analysis identified numbers of comorbidities in 21 patients in the Chadox1 cohort and two patients in the Ad26.COV2. S cohort as Meniere’s disease, Hashimoto’s thyroiditis, von Willebrand disease, hypertension, asthma, chronic neurologic disorders, and hypothyroidism as presented in Table 2.

### Thromboembolic events

In the 157 cases in the ChAdOx1 nCoV-19 group, most of the patients presented with various Thromboembolic events such as cerebral venous sinus thrombosis 18 (11.5%), cerebral venous thrombosis 15 (9.6%), deep vein thrombosis 20 (12.7%), and pulmonary embolism 16 (10.1%). The 16 cases in the Ad26.COV2. S group presented with cerebral venous sinus thrombosis 14 (87.5%), cerebral venous thrombosis 1 (6.3%), and pulmonary embolism 1 (6.3%) (Tables 1 and 2).

### Laboratory finding

The median level of platelet count was 33,500 cells/mm^3^ (7000–334,000) in 62 patients received ChAdOx1 nCoV-19 vaccine, and 20,000 cells/mm^3^ (9000–127,000) in 16 patients received Ad26.COV2 vaccine. The D-dimer levels in the ChAdOx1 nCoV-19 group were elevated in 52 patients (33.1%) while in Ad26.COV2. S group, it was elevated in 15 patients (93.7%). The antibodies against PF4 in the ChAdOx1 nCoV-19 group were positive in 39 patients (24.8%) while in Ad26.COV2. S group, it was positive in 13 patients (81.2%). The median fibrinogen level was 1.2 g/liter (0.4–5.7) in 62 patients who received the ChAdOx1 nCoV-19 vaccine and 141 (59–332) in 16 patients who received Ad26.COV2 vaccine. The detailed description of all laboratory findings can be seen in Tables 1 and 2.

### Treatment and Prognosis

In the 145 cases in the ChAdOx1 nCoV-19 group, 48 (30.6%) patients reported receiving one or more thrombosis treatment such as heparin therapy in 20 (41.6%), non-heparin anticoagulation in 8 (16.7%), corticosteroids in 18 (37.5%), and Intravenous immunoglobulin in 29 (60.4%). In Ad26.COV2. S group, all 16 patients reported receiving one or more treatment such as heparin therapy in 7 (43.8%), non-heparin anticoagulation in 2 (12.5%), and corticosteroids in 8 (50%).

In the ChAdOx1 nCoV-19 group, about 21.7% on the patients (n = 34) had achieved a full recovery, 13.4% (n = 21) were recovering, and 115.9% (n = 25) had died. While in Ad26.COV2. S group, about 28.5% on the patients (n = 4) had achieved a full recovery, 35.7% (n = 5) were recovering, and 21.4% (n = 3) had died. Of the 20 cases in the ChAdOx1 nCoV-19 group treated with heparin, eight patients reached full recovery, five patients were recovering, and six cases died. In patients treated with non-heparin anticoagulation, there were five cases of full recovery, one patient recovering, and no patient died. The prognostic and treatment details can be seen in Tables 1 and 2.

In the ChAdOx1 nCoV-19 group, 29 patients received IVIG, but only 8 patients reported prognoses with IVIG; five patients (17.2%) had achieved a full recovery, one patient (3.4%) was recovering, and two (6.9%) had died. We did not identify any published reports for IVIG withAd26.COV2. S vaccine.

### Quality assessment

**Fig. 2:**
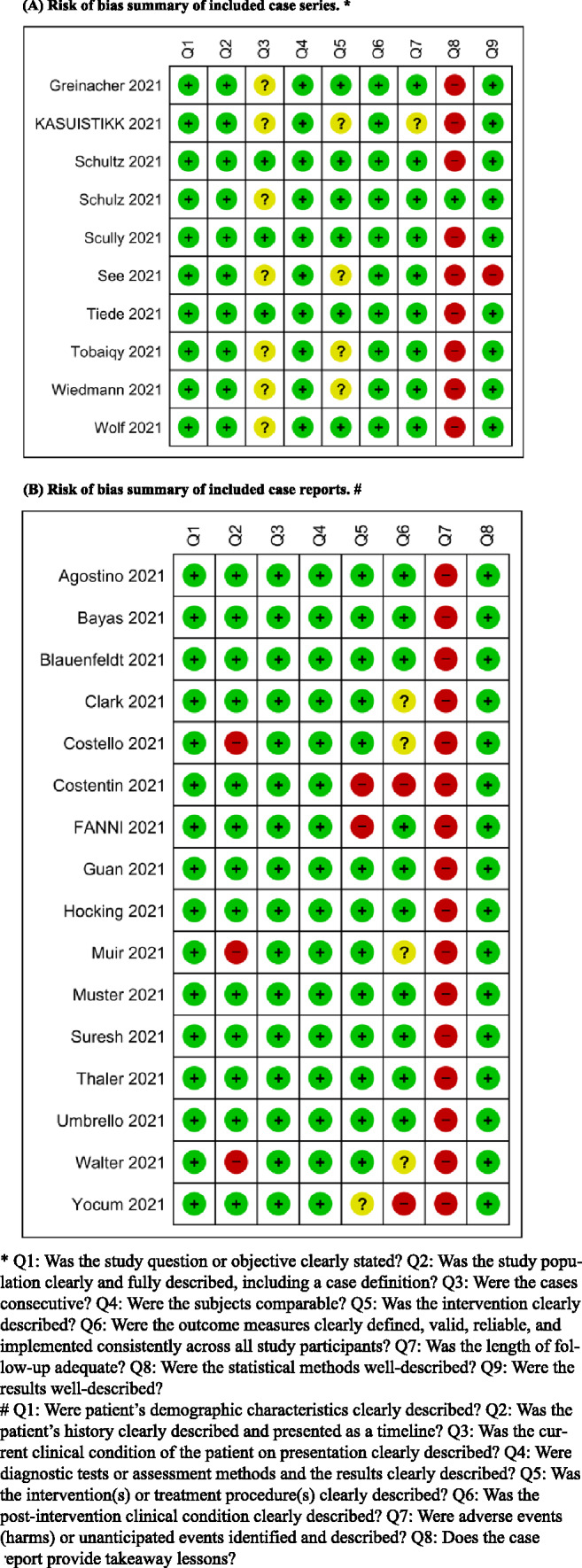
Shows the risk of bias summary for each included study: **A** Risk of bias summary of included Case series. **B** Risk of bias summary of included Case reports

Overall, the methodological quality of seven case reports was moderate to high quality according to the JBI Critical Appraisal Checklist for Case Reports [18]. According to NIH Quality Assessment Tool for Case Series Studies, the quality of the eight case series ranged from moderate to high quality [17]. The quality assessment of the included studies is shown in Fig. 2; the summary of the quality assessment of each study is shown in Supplementary S 1–2. The most concerning domains related to unclear statistical methods and unclear reporting on adverse events/conditions post-treatment.

## Discussion

In this systematic review of the published reports of VITT, we identified a total of 159 published cases, 145 of which occurred with the ChAdOx1 nCoV-19 vaccine, and 14 of which occurred with the Ad26.COV2. S vaccine. The Gam-COVID-Vac vaccine (recombinant adenovirus vector based on the human serotypes 3 and 26 adenovirus carrying the S-protein gene of the SARS-CoV-2) did not have any reports of VITT. The only mention of a thrombotic event with this vaccine was in a patient developing a DVT attributable to pre-existing comorbidities in one clinical trial [41].

Two important demographic features that stand out concerning these VITT events are age and sex distribution. The mean age for the events associated with the ChAdOx1 nCoV-19 vaccine was 43.5 years, and most events were associated with the Ad26.COV2. S vaccine occurred in those below 40 years of age. This age distribution has important public health implications, particularly with the vaccine-hesitancy rates on rise in these population age groups. Individuals in these middle age groups usually experience a milder COVID-19 than the elderly [42]; therefore, they might fearfully choose to avoid these vaccinations. Whenever feasible, health policymakers and public health officials may consider providing alternative vaccines to those at the highest risk of VITT.

It is worth our notice that some individuals may be at a higher risk of COVID-induced thrombotic events, as cases of coagulopathy have been reported in younger individuals [43, 44]. Nevertheless, the risk–benefit calculus is likely to vary depending on an individual’s age, local infection rates, and comorbidities. Indeed, in the UK, those below the age of 30 have been offered alternative vaccination options due to similar concerns [45]. This is particularly important from a global health perspective in those developing countries, which tend to have younger populations, may be more affected by this issue.

An important positive aspect of our findings is that elderly patients, who are at higher risk of COVID-related complications [46] and most in need of vaccines, seemed less likely to suffer from VITT based on our data since most cases occurred in younger individuals.

In addition, there was a relative preponderance of females with VITT in this study (73% in ChAdOx1 nCoV-19 vaccine, and all the cases in Ad26.COV2. S vaccine). Although there may be some concerns that the higher number of females with events is merely a reflection of more females than males getting vaccinated, studies do not suggest the discrepancies in vaccination rates, which are often in the 40 vs. 60%-range [47], fully account for this difference. However, it is important to note that females are generally more likely to seek healthcare services than males [48], which may be an additional contributing factor.

Another issue is that the cases in our review had a relatively low number of comorbidities. However, our study design does not allow us to determine whether any of the comorbidities mentioned above is a risk factor for thrombotic events. Since healthy individuals are relatively less likely than individuals with several comorbidities to have COVID-19-related complications, they may be tempted to avoid these vaccines under the possibly erroneous assumption that they are less likely to die from COVID-19 than from a vaccine-associated thrombotic event.

Regarding the clinical presentation of these patients, the most common category of symptoms was neurological in nature, including headaches, seizures, hemiparesis, and ophthalmic symptoms. Nevertheless, it is important to note that various additional symptoms may be present, including fever, cough, epigastric pain, and fatigue. Therefore, the sensitivity of any set of symptoms may not be very high, although this would require confirmation in future studies.

These symptoms are distributed following the course of venous thrombi, most of which occur within the cerebral venous system. That said, it is important to note that thrombi may be detected in other locations, such as the lower limb veins (leading to pulmonary emboli) and the portal veins. These fewer common locations for thrombus formation may account for the less commonly presenting symptoms such as cough and abdominal pain.

The theorized mechanism behind VITT has been closely linked to that of heparin-induced thrombotic Thrombocytopenia (HIT). Supporting data for this hypothesis include the presence of anti-platelet factor 4 antibodies (anti-PF4) in both conditions [49]. These antibodies then bind to platelets, creating immune complexes and precipitating thrombotic events. In their study, Greinacher et al. [49] propose the following mechanism: following injection, vaccine components activate platelets, which release PF4. Vaccine components then bind to PF4, creating an immunogenic substance that is attacked by circulating IgG. Consequently, this forms a PF4/IgG complex that can bind to the surface of platelets and activate them, resulting in a prothrombotic state. Notably, previous data on mice models did show thrombocytopenia as a transient adverse effect following recombinant adenovirus-vectors [50].

However, our study points out one clinical difference between VITT and HIT. Unlike HIT, where DVT is usually the predominant thrombotic manifestation [51], our study shows cerebral thrombi to be more common overall, although 13.8% of patients had a DVT. Nevertheless, we find similarities with HIT as well. The average time from vaccination to admission amongst our studies was 10.9 and 15.2 days with the ChAdOx1 nCoV-19 and Ad26.COV2.S vaccines, respectively, which approximately corresponds to the temporal pattern of HIT, are typically quoted as occurring after 5 to 14 days post-exposure to heparin [52, 53]. Our findings were relatively close to previous studies; in a cohort study of 170 patients who developed VITT after receiving the ChAdOx1 nCoV-19 vaccine, 97% of patients presented to the hospital with symptoms with 5th to 30th day with an average of 13.5 days from vaccination [54]. A systematic review that included data of 41 patients reported average durations from vaccination to admission of 8.9 and 10.3 days for the ChAdOx1 nCoV-19 and Ad26.COV2.S vaccines respectively [55].

Also in accordance with the clinical picture of HIT, where bleeding is very rare, is the absence of bleeding reports amongst our included reports [51]. This is despite the thrombocytopenic state common to both conditions. Importantly, the median platelet count in our study was 34,000 in the ChAdOx1 nCov-19 cohort, which points to a more vigorous platelet-depleting process than HIT, where platelets usually remain above 50,000 [56].

In our review, neither PT nor PTT was markedly prolonged, and Hb was not significantly decreased. In contrast, D-dimers seemed to be a relatively more sensitive marker amongst the included patients. Among those with anti-PF4 measured, most had positive titers, which corresponds with the findings mentioned above by Greinacher et al. [49]. Generally, ELISA anti-PF4 assays are recommended over other measurement modalities [39], as they may have higher sensitivity. Nevertheless, even anti-PF4 may not be 100% sensitive, as there was one negative anti-PF4 ELISA measurement in our study.

Since VITT is a relatively novel phenomenon, there are currently no robust evidence-based treatments with proven efficacy; however, given the similarities to HIT, similar treatment paradigms have been advocated [53, 57, 58]. Namely, the use of IVIG and direct anticoagulants such as argatroban or bivalirudin has been suggested in the acute phase, whereas the use of heparin products has been discouraged [57]. Further management would include eventual transition to an oral anticoagulant, preferably a DOAC rather than a vitamin-K-based regimen [53]. However, in our sample, 12.4% of patients received heparin products. The similarities to HIT in terms of pathophysiology caution that the use of heparin products may be unadvisable. IVIG and steroids were also utilized in some patients (6.2 and 9.7% respectively in the ChAdOx1 nCov-19 cohort). After IVIG, five patients (17.2%) had achieved a full recovery, one patient (3.4%) was recovering, and two (6.9%) had died. IVIG is recommended to improve VITT cases as IVIG inhibits the hypercoagulability process and increases platelet count, which in turn reduces the severity of VITT [59, 60]. The American Heart Association recommended IVIG 1 g/kg for 2 days after testing positive for antibodies against PF4 [55]. To the best of our knowledge, there is no current evidence to support the use of steroids in HIT, with one observational study reporting an adverse association [61]. Generally speaking, platelet transfusions are advised against [57]; however, the American Association of Hematology currently states that platelet transfusions may be used if the condition is refractory to other modes of treatment and life-threatening bleeding is occurring [62]. Nevertheless, further studies are needed to understand whether treatments typically used in HIT patients can be safely and effectively extrapolated to VITT, as the evidence base for this entity is lacking.

Regarding the outcomes of patients with VITT, many patients in our study had recovered or were recovering; however, there was a relatively high case-fatality rate (13.8 and 21.4% in the ChAdOx1 nCov-19 and Ad26.COV2. S groups, respectively). This is likely an overestimate of the fatality rate as the cases most likely to be reported in the literature are ones on the severe end of the spectrum.

There are several important limitations of our study: First, as with any other review of the literature, there may be the concern of publication bias, as not all cases of VITT are equally likely to be published. Second, the available literature may have included the subset of patients with the most severe presentations of VITT, as milder cases may have been less likely to seek medical care; therefore, we cannot readily generalize the representativeness of our results across all possible cases of VITT. Third, though comorbidities were uncommon in our study population, our study design does not allow us to definitively establish an association (or lack thereof) between VITT and underlying comorbidities. Fourth, some included studies did not report all lab measurements, which may limit the accuracy of our findings.

**Fig. 3:**
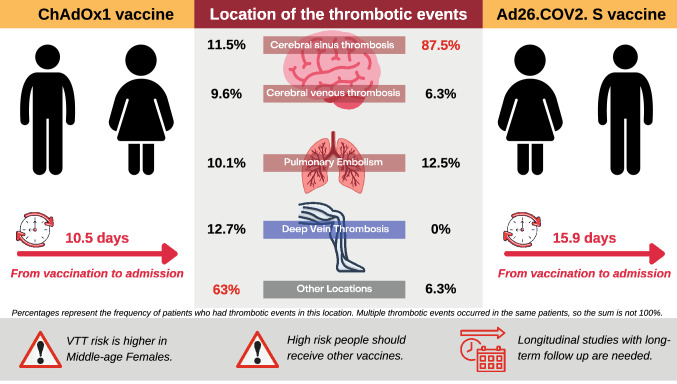
The take home message for clinicians

Finally, the take-home message from this study for clinicians is that: (1) VITT might occur in young individuals and particularly females, (2) the typical presentation of VITT might include cerebral thrombi and other heterogeneous events, therefore, clinical experience is important for detection and early management of VITT in the vaccinated individuals, and (3) for individuals who are at higher risk of VITT, alternative SARS-CoV-2 vaccines should be provided whenever possible (Fig. 3). For future research, we recommend further large long-term longitudinal studies of the individuals who received ChAdOx1 nCov-19 and Ad26.COV2. S vaccines with the aim of identifying the magnitude and risk factors of VITT in the different age groups. Such information will be important to inform health policy makers to guide their decisions about population vaccination choices.

In conclusion, in this study, we described the certain demographics associated with VITT and the clinical presentations of those cases in the ChAdOx1 nCoV-19 and Ad26.COV2. S vaccines. Young individuals, particularly females, may be more susceptible to VITT, and future studies should seek to confirm this association. In addition, the clinical presentation of VITT commonly includes cerebral thrombi, pulmonary embolism, and deep venous thrombosis, but other presentations are also possible, highlighting the importance of clinical vigilance in recent vaccine recipients. Finally, the coagulation profile does not seem to be markedly altered in patients with VITT, with D-dimers and anti-PF4 likely being the most sensitive.

## Supplementary Information

Below is the link to the electronic supplementary material.

Supplementary file1 (DOCX 36 KB)
