# Supplementary material for: A systematic review of vaccine-induced thrombotic thrombocytopenia in individuals who received COVID-19 adenoviral-vector-based vaccines
Source: J Thromb Thrombolysis. 2022 Feb 14;53(4):798–823. doi: 10.1007/s11239-021-02626-w (PMC8853120; doi:10.1007/s11239-021-02626-w)
Supplement: Supplementary file 1 — Supplementary file1 (DOCX 36 KB) [file 11239_2021_2626_MOESM1_ESM.docx]

**Search strategy:**

1. (“ChAdOx1 nCoV-19 vaccine” OR “AstraZeneca Vaccine”).
2. (“Ad26.COV2. S” OR “Johnson & Johnson Vaccine”).
3. (COVID-19 OR “Disease, COVID-19 Virus” OR “Virus Disease, COVID-19” OR “COVID-19 Virus Infection” OR “Infection, COVID-19 Virus” OR “Virus Infection, COVID-19” OR “2019-nCoV Infection” OR “2019 nCoV Infection” OR “2019-nCoV Infections” OR “Infection, 2019-nCoV” OR “Coronavirus Disease-19” OR “Coronavirus Disease 19” OR “2019 Novel Coronavirus Disease” OR “2019 Novel Coronavirus Infection” OR “2019-nCoV Disease” OR “2019 nCoV Disease” OR “2019-nCoV Diseases” OR “Disease, 2019-nCoV” OR “COVID19” OR “SARS Coronavirus 2 Infection” OR “SARS-CoV-2 Infection” OR “Infection, SARS-CoV-2” OR “SARS CoV 2 Infection” OR “SARS-CoV-2 Infections” OR “Pandemic, COVID-19).
4. #1 AND #3.
5. #2 AND #3.
6. #4 AND #5.
7. Limit #6 from December 2020 till May 2021

Table1.

| Study ID | NIH Quality Assessment Tool for Case Series Studies Criteria Met | | | | | | | | | Quality (Total Quality Score) |
| --- | --- | --- | --- | --- | --- | --- | --- | --- | --- | --- |
|  | Was the study question or objective clearly stated? | Was the study population clearly and fully described, including a case definition? | Were the cases consecutive? | Were the subjects comparable? | Was the intervention clearly described? | Were the outcome measures clearly defined, valid, reliable, and implemented consistently across all study participants? | Was the length of follow-up adequate? | Were the statistical methods well-described? | Were the results well-described? |  |
| Greinacher 2021 | YES | YES | NA | YES | YES | YES | YES | NO | YES | Good (7) |
| KASUISTIKK 2021 | YES | YES | NA | YES | NA | YES | NA | NO | YES | Fair (5) |
| Schultz 2021 | YES | YES | YES | YES | YES | YES | YES | NO | YES | Good (8) |
| Schulz 2021 | YES | YES | NA | YES | YES | YES | YES | YES | YES | Good (8) |
| Scully 2021 | YES | YES | YES | YES | YES | YES | YES | NO | YES | Good (8) |
| See 2021 | YES | YES | NA | YES | NA | YES | YES | NO | NO | Fair (5) |
| Tiede 2021 | YES | YES | YES | YES | YES | YES | YES | NO | YES | Good (8) |
| Tobaiqy 2021 | YES | YES | NA | YES | NA | YES | YES | NO | YES | Good (6) |
| Wiedmann 2021 | YES | YES | NA | YES | NA | YES | YES | NO | YES | Good (6) |
| Wolf 2021 | YES | YES | NA | YES | YES | YES | YES | NO | YES | Good (7) |

Table (1) shows a quality Assessment of Included case series Studies using NIH Quality Assessment Tool. NA: not applicable.

Table 2.

| Study ID | The Joanna Briggs Institute (JBI) Critical Appraisal checklist for Case Reports | | | | | | | | Quality (Total Quality Score) |
| --- | --- | --- | --- | --- | --- | --- | --- | --- | --- |
|  | Were patient’s demographic characteristics clearly described? | Was the patient’s history clearly described and presented as a timeline? | Was the current clinical condition of the patient on presentation clearly described? | Were diagnostic tests or assessment methods and the results clearly described? | Was the intervention(s) or treatment procedure(s) clearly described? | Was the post-intervention clinical condition clearly described? | Were adverse events (harms) or unanticipated events identified and described? | Does the case report provide takeaway lessons? |  |
| Agostino 2021 | Yes | Yes | Yes | Yes | Yes | Yes | NA | Yes | 88% |
| Bayas 2021 | Yes | Yes | Yes | Yes | Yes | Yes | NA | Yes | 88% |
| Blauenfeldt 2021 | Yes | Yes | Yes | Yes | Yes | Yes | NA | Yes | 88% |
| Clark 2021 | Yes | Yes | Yes | Yes | Yes | Unclear | NA | Yes | 81% |
| Costello 2021 | Yes | NA | Yes | Yes | Yes | Unclear | NA | Yes | 69% |
| Costentin 2021 | Yes | Yes | Yes | Yes | NA | NA | NA | Yes | 63% |
| Fanni 2021 | Yes | Yes | Yes | Yes | NA | Yes | NA | Yes | 75% |
| Guan 2021 | Yes | Yes | Yes | Yes | Yes | Yes | NA | Yes | 88% |
| Hocking 2021 | Yes | Yes | Yes | Yes | Yes | Yes | NA | Yes | 88% |
| Muir 2021 | Yes | NA | Yes | Yes | Yes | Unclear | NA | Yes | 69% |
| Muster 2021 | Yes | Yes | Yes | Yes | Yes | Yes | NA | Yes | 88% |
| Suresh 2021 | Yes | Yes | Yes | Yes | Yes | Yes | NA | Yes | 88% |
| Thaler 2021 | Yes | Yes | Yes | Yes | Yes | Yes | NA | Yes | 88% |
| Umbrello 2021 | Yes | Yes | Yes | Yes | Yes | Yes | NA | Yes | 88% |
| Walter 2021 | Yes | NA | Yes | Yes | Yes | Unclear | NA | Yes | 69% |
| Yocum 2021 | Yes | Yes | Yes | Yes | Unclear | NA | NA | Yes | 69% |

Table (2) shows a quality Assessment of Included case reports Studies using The Joanna Briggs Institute (JBI) Critical Appraisal checklist. NA: not applicable.
